# The efficacy of the “Talk-to-Me” suicide prevention and mental health education program for tertiary students: a crossover randomised control trial

**DOI:** 10.1007/s00787-022-02094-4

**Published:** 2022-10-04

**Authors:** Bahareh Afsharnejad, Ben Milbourn, Maya Hayden-Evans, Ellie Baker-Young, Melissa H. Black, Craig Thompson, Sarah McGarry, Melissa Grobler, Rhonda Clifford, Frank Zimmermann, Viktor Kacic, Penelope Hasking, Sven Bölte, Marcel Romanos, Tawanda Machingura, Sonya Girdler

**Affiliations:** 1 School of Allied Health, Curtin University, Perth, WA Australia; 2 Curtin Autism Research Group (CARG), Curtin University, Perth, WA Australia; 3 enAble Institute, Curtin University, Perth, WA Australia; 4 Faculty of Health and Medical Sciences, The University of Western Australia, Perth, WA Australia; 5 Klinikum Aschaffenburg Hospital for Child and Adolescent Psychiatry and Psychotherapy, Aschaffenburg, Germany; 6 Center of Neurodevelopmental Disorders (KIND), Centre for Psychiatry Research, Department of Women’s and Children’s Health, Karolinska Institutet & Stockholm Health Care Services, Region Stockholm, Stockholm, Sweden; 7 Child and Adolescent Psychiatry, Stockholm Health Care Services, Region Stockholm, Stockholm, Sweden; 8 Department of Child and Adolescent Psychiatry, Psychosomatics and Psychotherapy, University Hospital Würzburg, Würzburg, Germany; 9 Faculty of Health Sciences & Medicine, Bond University, Queensland, Australia; 10 Center of Neurodevelopmental Disorders (KIND), Centre for Psychiatry Research, Division of Neuropsychiatry, Department of Women’s and Children’s Health, Karolinska Institutet & Child and Adolescent Psychiatry, Stockholm Health Care Services, Stockholm County Council, Stockholm, Sweden; 11 School of Population Health, Curtin University, Perth, WA Australia

**Keywords:** Suicide prevention program, Mass open online course (MOOC), Mental health education, University students

## Abstract

Despite suicide ideation being one of the most frequently reported health issues impacting tertiary students, there is a paucity of research evaluating the efficacy of preventive interventions aimed at improving mental health outcomes for students studying at two tertiary institutes. The current study evaluated the efficacy of the “Talk-to-Me” Mass Open Online Course (MOOC) in improving tertiary students’ abilities to support the mental health of themselves and their peers via a randomised controlled trial design, comparing them to a waitlist control group. Overall, 129 tertiary students (*M* = 25.22 years, *SD* = 7.43; 80% female) undertaking a health science or education course at two Western Australian universities were randomly allocated to either “Talk-to-Me” (*n* = 66) or waitlist control (*n* = 63) groups. The participants’ responses to suicidal statements (primary outcome), knowledge of mental health, generalised self-efficacy, coping skills, and overall utility of the program (secondary outcomes) were collected at three timepoints (baseline 10-weeks and 24-weeks from baseline). Assessment time and group interaction were explored using a random-effects regression model, examining changes in the primary and secondary outcomes. Intention-to-treat analysis (*N* = 129) at 10-weeks demonstrated a significant improvement in generalised self-efficacy for “Talk-to-Me” compared to the control group (ES = 0.36, *p* = .04), with only the “Talk-to-Me” participants reporting increased knowledge in responding to suicidal ideation (primary outcome). This change was sustained for 24 weeks. Findings provide preliminary evidence suggesting that the “Talk-to-Me” MOOC can effectively improve tertiary students’ mental health and knowledge of how to support themselves and others in distress. ACTRN12619000630112, registered 18-03-2019, anzctr.org.au.

**Supplementary Information:**

The online version contains supplementary material available at 10.1007/s00787-022-02094-4.

## Introduction

Young adulthood represents a period of necessary personal growth and development, moving towards mastering the increased demands of adulthood and developing a personal identity [1]. The assimilation of adult roles and the challenges of balancing work, study and social commitments increase the risk of young adults experiencing mental health challenges [2]. Prior to the onset of the COVID-19 pandemic, reports indicated that between 13 and 20% of Australian young adults, including those studying at a tertiary level, were experiencing psychological distress related to academic and financial pressures, isolation, loneliness and poor self-care [3, 4].

In 2019, a total of 1,609,798 students were studying at the tertiary level in Australia [5], with 65% reported experiencing mental health issues (e.g., anxiety and mood disorders) [6]. The onset of the COVID-19 pandemic in March 2020 necessitated changes to the mode of delivery for tertiary education in Australia, with the majority of classes moving to an online format [7]. Further, COVID-19 negatively impacted the mental health of Australian tertiary students [8], who experienced high levels of social isolation and loss of casual employment, increasing their vulnerability to self-harm and suicide ideation [9].

Suicide is a leading cause of death among young adults, accounting for an estimated 700,000 deaths globally each year [10]. The rate of suicidal ideation among tertiary students is disproportionately high compared to the general public and remains a major concern for universities [11]. Despite the high prevalence of mental health concerns among this population, help-seeking behaviour is low, likely as a result of the continued stigma surrounding mental health or/and a lack of easily accessible, low threshold supports [12]. Instead, young adults commonly confide in their peers when experiencing mental health difficulties, who are often their sole source of support [13]. While youth believe in the importance of suicide prevention strategies, in the presence of suicidal ideation warning signs and risk factors, they rarely intervene [14]. Evidence suggests that this lack of action stems largely from young adults’ poor self-efficacy in identifying warning signs, missing opportunities for intervention, and preventing suicide attempts and the loss of life [14]. Given that tertiary students are time-poor with limited resources, engaging them in Suicide prevention programs is challenging [15]. Targeted online Suicide prevention programs, being self-paced, easily accessible, and relatively low cost, may mitigate many of these barriers [16].

Recently, a literature review exploring Suicide prevention programs targeting tertiary students [17] highlighted that the majority of programs were directed at gatekeepers rather than tertiary students themselves and delivered almost exclusively face-to-face. While overall findings across studies indicated that these training programs might effectively improve mental health knowledge and attitudes, student outcomes were rarely measured. Therefore, little is known of the role of prevention programs in impacting student engagement with services, their suicidal ideation or broader mental health outcomes [17]. While increasingly tertiary education is delivered via virtual means, pointing to an opportunity for delivering suicide programs online [18], to date, only one such program has evaluated tertiary students increased knowledge of prevention strategies two months post completion of the program [19, 20], with the application of this knowledge in real-life situations remaining unknown. Additionally, evaluations of gatekeeper programs fail to assess or demonstrate sustained improvement on self-report outcome measures [20].

The “Talk-to-Me” program is a Suicide prevention program originating in Germany that aims to teach students to identify and respond to suicidal crises and suicidal thoughts and behaviours. The program has recently been adapted for an Australian context, delivered online as a Massive Open Online Course (MOOC), a course of study made freely available over the internet to a very large number of people. The MOOC consists of six modules: (1) Mental fitness; (2) Strategies to increase mental fitness; (3) Self-injury; (4) Suicidal behaviour in young adults; (5) Interventions for suicidal behaviour; and (6) Gatekeeper interventions. The “Talk-to-Me” MOOC aims to raise awareness of mental health-promoting activities, build resilience, and equip students with practical skills to deal with their own and others’ mental distress. The online delivery format alleviates some of the barriers (e.g., lack of time and financial security) [11] to engaging tertiary students, allowing continuity in delivery in the context of government lockdowns in response to COVID-19. Given the paucity of research evaluating the efficacy of online Suicide prevention programs aiming to improve responding to suicidal thoughts/behaviour of tertiary students [21], a crossover RCT design was employed to evaluate the efficacy of an online version of the “Talk-to-Me” program in improving tertiary students’ abilities to support someone experiencing suicidal thoughts.

## Methods

### Design

Following a pre-defined protocol [22] and in line with the CONSORT guidelines [23], this study employed a pragmatic classic crossover RCT design to evaluate the efficacy of an online mental health education and suicide awareness program (“Talk-to-Me”). The factors within this crossover design included Group (MOOC vs treatment as usual) and Periods (before and after the crossover). Each period of the study was aligned with one Western Australian University semester, running for 10 weeks. It was anticipated that the allocated time would allow students enough time to complete the 6 modules of the “Talk-to-Me” MOOC, considering their study and work commitments. The two periods of the study were divided by a 4-week university semester break. A sample of 129 university students was randomly assigned to either the early start group (ESG: receiving the MOOC in the first period) or the delayed start group (DSG: receiving the MOOC in the second period). Notably, the first period of the study (weeks 2–6) coincided with the COVID-19 global pandemic, with strict ‘lockdown’ restrictions implemented in Western Australia from the 15th of March and easing towards the end of April 2020. During this time, most university tutorials, workshops, and labs were delivered online. The MOOC was delivered via an online learning destination provided by the edX platform [24]. Data were collected from both ESG and DSG students at three-time points: (T0) baseline (before randomisation and in the same session as screening); (T1) Upon completion of the first period (after week 10); and (T2) Upon completion of the second period (after week 24). All data collection, management and coordination, were undertaken at Curtin University in Perth, Western Australia.

### Participants

Participants were recruited via flyers sent through the university learning management systems of two universities in Perth, Western Australia (Curtin University and the University of Western Australia) from February to March 2020. Full or part-time students enrolled in health or education degrees from these universities enrolled in a second or third-year undergraduate or Graduate Entry Master's level were eligible to participate in the study. First and fourth-year undergraduate students were not invited due to the potential peak of study-related anxiety or stress levels [25]. Following the obtaining of informed consent, the suicidal ideation of participants was screened via the Suicidal Ideation Attributes Scale (SIDAS) [26]. Students not registering on the edX platform and those scoring above 21 or between 7 and 10 on items assessing suicide attempts as assessed by SIDAS were excluded from the present study, with the latter referred to their treating clinician or appropriate services and support for follow-up. As a token of appreciation for volunteering their time, students received a certificate for completing the MOOC and went into a draw for coffee vouchers.

### Intervention

“Talk-to-Me” is an online psychoeducational Suicide prevention program targeting young adults [27]. The program was adapted from an earlier version developed in Germany, collaborating with clinical experts and the German Suicide Prevention Program (World Health Organization). This skills training program aims to increase young adults' awareness of mental health-promoting activities, improve their resilience, develop their distress management skills and ability to identify the early signs of suicide ideation or behaviour in themselves and others and apply suicide crisis intervention strategies. The six modules of the “Talk-to-Me” MOOC were translated and adapted to Australian English through a co-production process with stakeholders (health service consumers, university students, not-for-profit and government-funded mental health services), enhancing its quality and relevance for Australian tertiary institution students. Mental health education principles, such as the PERMA framework [28], were incorporated into the modules, with two scenarios presented at the end of each module, with the goals of increasing students’ awareness of their own and others’ mental health needs and support them in developing practical skills to respond to self-injury and suicidal ideation (Online Resource 1).

### Procedures

Prior to group allocation, a survey link to Qualtrics [29] was sent to all participants in ESG and DSG, requesting their screening and baseline data. Data from the screening survey provided evidence for the eligibility of the participants as assessed via SIDAS [26]. The survey also gathered participants' sociodemographic information, including their age, gender, ethnicity, university, year level, highest education level, mode of study (full-time/part-time), course of study, previous mental health training experience and postcode (as a proxy of the socioeconomic status). After the initial screening, those meeting the eligibility criteria were then allocated to ESG or DSG using 1:1 online simple randomisation. The primary and secondary outcome measures were collected at baseline (T0) and after weeks 10 (T1) and 24 (T2). The SIDAS scores were collected once more before the commencement of Period 2 only for those allocated to DSG to reassess their eligibility. As the modules were delivered online, the only information available about the participants’ dosage was whether they commenced the “Talk-to-Me” modules.

#### Primary outcome measure

*Suicide intervention response inventory—version 2 (SIRI-2)* is a 24-item self-report measure evaluating the participants’ ability to identify and appropriately respond to suicidal statements [30]. This measure represents realistic quotes from hypothetical suicidal clients, with each statement paired with two corresponding hypothetical responses, yielding 48 responses. The SIRI-2 is rated on a 7-point Likert scale, ranging from +3 (*highly appropriate*) to − 3 (*highly inappropriate*). The scores were calculated by summing the absolute difference between each item’s score and a corresponding score derived from a sample of clinicians, with lower scores indicating less divergence from clinicians’ ratings and thus demonstrating more appropriate crisis communication skills [30]. SIRI-2 has demonstrated good reliability of over .9 and great sensitivity to suicide counselling programs [30].

#### Secondary outcome measures

*The objective structured video examination (OSVE)* was developed based on the work of Selim and Dawood [31] specifically for this study, assessing the extent to which the participants had assimilated the MOOC’s content (Online Resource 2). The measure contains five 2–5-minute videos followed by five 5-response multichoice questions. The video scenarios were aligned with the content of the MOOC modules, focusing on a student struggling with his mental health, with questions ascertaining participants’ abilities in identifying risk factors and negative thinking patterns in responding to self-harm, recommending coping strategies, and developing a safety plan in the presence of suicide risk (e.g. “*Which of the following are all suicide risk factors for Mike*?” or “*Which are all three key components to creating a safety plan for Mike*?”). A score of one was awarded to each right answer. The total score for the OSVE measure range from 0 to 25, with higher scores indicating greater learning from the MOOC content. Despite the novelty of the measure for this study, the OSVE format has been previously used as a tool in evaluating learning in response to a science course [31], demonstrating acceptable reliability (Cronbach’s *α* = 0.71) and concurrent validity (*r *= 0.60) [32].

*The brief resilience scale (BRS)* is a 6-item self-report measure assessing participants’ perceived ability to recover from stress and any adverse events they have experienced [32, 33]. For example, *“I tend to bounce back quickly after hard times”*. The BRS is rated on a 5-point Likert scale, with scores ranging from 1 (*strongly disagree*) to 5 (*strongly agree*). The BRS total score ranges from 6 to 30, with higher scores indicating greater resilience [33]. The measure has shown excellent reliability (Cronbach’s *α* = [0.8, 0.91]) and an acceptable concurrent validity [33, 34].

*Perception of academic stress scale (PASS)* is an 18-item self-report measure evaluating perceived academic stress and its causes [35]. For example, *“I am unable to catch up if I am getting behind the work”.* The measure is rated on a 5-point Likert scale, ranging from 1 (*strongly disagree*) to 5 (*strongly agree*) [35]. The PASS total scores range from 18 to 90, with higher scores indicating greater perceived academic stress. PASS not only has shown good reliability (Cronbach’s *α* = 0.90), but it also has evidence for face, content and convergent validity among university students [35].

*General self-efficacy scale (GSE)* is a 10-item self-report assessing perceived generalised self-efficacy [36]. For example, “*If I am in trouble, I can usually think of a solution”.* The measure is rated on a 4-point Likert scale, ranging from 1 (*not true at all*) to 4 (*exactly true*). The GSE total scores range from 10 to 40, with higher total scores indicating greater self-efficacy. GSE has been evaluated across many contexts, demonstrating good validity (*r*=.67) and reliability with Cronbach’s *α* ranging from 0.75 to 0.91 [36].

*Attitudes towards seeking professional psychological help scale (ATSPPHS-SF)* is a 10-item self-report measure exploring the participants’ attitudes towards help-seeking behaviours when experiencing mental health issues 77 [37]. For example, “*If I believed I was having a mental breakdown, my first inclination would be to get professional attention”*. The measure is rated on a 4-point Likert scale, ranging from 0 (*disagree*) to 3 (*agree*). The total scores range from 0 to 30, with higher scores indicating more positive attitudes towards seeking help from professionals [37]. This widely used measure of help-seeking attitudes has shown good reliability with Cronbach’s *α* of 0.77 [38].

*MOOC questionnaire* was developed for this study to explore the participants’ experiences and satisfaction with the MOOC (Online Resource 3). The questionnaire was delivered in both multiple-choice and text entry formats. The multiple-choice component consisted of two sections enquiring about the participants’ satisfaction with the content and their perception of how helpful and engaging the program was. Satisfaction with the content was assessed via 13 questions rated on a 5-point Likert scale ranging from 0 (*Not at all*) to 4 (*Very*), with higher scores indicating higher satisfaction levels. Helpfulness was assessed via 14 questions rated on a 4-point Likert scale ranging from 0 (*Not helpful at all*) to 4 (*Very helpful*), with higher scores indicating the participants found the program more helpful. Data were collected at T1 for ESG immediately following participants' completion of the ‘Talk to Me’ MOOC.

*COVID-19 survey* was developed for this study and collected at T1 due to its co-occurrence with the onset of the COVID-19 global pandemic (Online Resource 4). This 31-item survey explored the impact of COVID-19 on the participant’s everyday life and mental health. For example, “Since COVID-19, I feel more burdened”. The measure consists of three dichotomous items (*Yes*=1; *No*=0) and 28 items scored on a 4-point Likert scale, ranging from 0 (*Never*) to 3 (*Always*). Some items in the measure are reverse scored (Online Resource 4). Total scores ranged from 0 to 87, with higher scores indicating a more negative impact.

### Statistical analysis

A data frame with 300 participants allocated to intervention and control groups and assessed at three assessment time points was created using RStudio Version 4.2.1 [39]. First the appropriate model (y ~ group + time +group*time + (1|id) + ɛ) was fitted (lme4 package [40]). Then power estimates were obtained at different sample sizes using the simr packages [41]. Based on multiple power analyses, a sample size of at least 120 students might suffice to obtain a power of > 80%. To Account for the negative influence of factors decreasing the power of the study, it was anticipated to recruit a larger sample of 170 students for this study. Using chi-square (categorical variables) or independent samples t-tests/Mann-Whitney *U* tests (continuous/ordinal variables), comparability of the ESG and DSG groups was explored at T0. Upon testing for the normality of the data, utilising the SPSS version 24 statistical software [42], the random-effects regression model was employed to explore the impact of the independent variables (fixed effects) such as assessment time (T0/T1), group (ESG/DSG) and sociodemographic data (gender, age, education, course, etc.) on the dependent variables (primary/secondary outcome measures). Using the participants’ identification codes (random effect) enabled an estimation of the correlation between measurements for each participant across the study period (time by group interaction). To explore possible moderation effects, a three-way interaction of time by group by other independent variables was conducted. The Bonferroni correction was employed to control for familywise error rates arising from multiple comparisons. Changes in the scores were considered significant if the results indicated a *p* < 0.05. Missing data were imputed based on the intent-to-treat (ITT) approach, enabling data comparison with and without ad-hoc imputation [43]. Cohen’s d Effect size was calculated using free online software by *Psychometrica* [44], with effect sizes 0.3, 0.5, and 0.8 indicating small, medium and large effects, respectively.

## Results

### Study flow

**Fig. 1:**
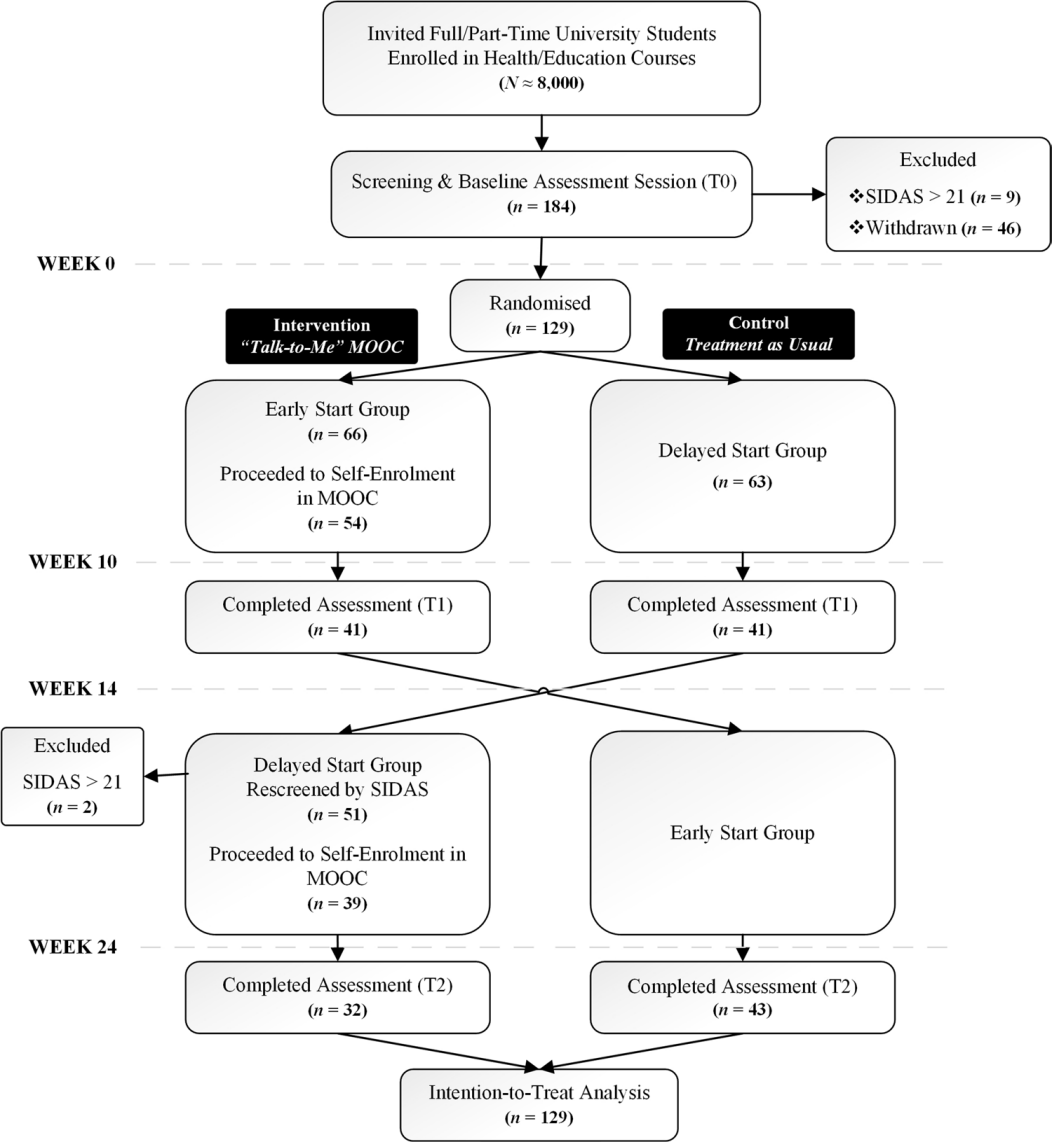
Participant recruitment, allocation and assessment Process based on the CONSORT diagram [23]

As demonstrated in Figure 1 from a pool of over 8,000 students from Curtin University and the University of Western Australia, 184 students participated in the screening. Nine students were excluded having SIDAS scores greater than the threshold of 21. A further 46 participants, although taking part in the screening session, did not progress to enrolling on the EdX platform and hence were excluded from the study. The remaining students (*N* = 129) were randomised to either ESG (*n* = 66) starting in Semester 1 or DSG (*n *= 63) starting in Semester 2 after a 4-week break. At 10 weeks, from each group, 12 students (ESG = 18%; DSG = 19%) were lost due to attrition, with 41 students (62%) from ESG and 51 (81%) from DSG completing the T1 assessment.

Overall, of the 66 students in ESG, 12 (18%) did not proceed to enroll in the MOOC. A comparison between those who did or did not self-enroll in the MOOC demonstrated that the latter group was predominantly younger (*p* = 0.01), male or non-binary (*p* = 0.006), and of a higher socioeconomic status (*p* = 0.03).

The comparison of the sociodemographics, education background and outcome measures of the participants demonstrated no significant difference between ESG and DSG at baseline. The participants from the ESG group were predominantly female (82%) with an average age of 24.75 years (SD = 7.11), studying at either Curtin University (89%) or the University of Western Australia (11%), mostly at an undergraduate level (Undergraduate 3rd year students = 50%; Undergraduate 2nd year students = 30%). Similar to the ESG, more than three-quarters of the DSG students were female (78%) with an average age of 25.70 years (SD = 7.80), with the majority studying at Curtin University (92%). The students studied mostly at an undergraduate level (Undergraduate 2nd year students = 36%; Undergraduate 3rd year students=37%). Table 1 demonstrates the demographic information for ESG and DSG, and Table 2 shows the means and standard deviation at each assessment time.

**Table 1:** Participants’ demographics and clinical characteristics

Students	ESG (*n* = 66)	DSG (*n* = 63)
Age (years), mean (SD)	24.75 (7.11)	25.70 (7.80)
Range	18–48 years	18–49 years
SIDAS^b^, mean (SD)	4.30 (6.17)	4.48 (6.47)
Gender, *n* (%)		
Female	54 (82%)	49 (78%)
Male	11 (17%)	13 (20%)
Non-binary/prefer not to say	1 (1%)	1 (2%)
Year level, *n* (%)		
Postgraduate student	13 (20%)	17 (27%)
Undergraduate 3rd-year students	33 (50%)	22 (35%)
Undergraduate 2nd-year students	20 (30%)	23 (36%)
University, *n* (%)		
Curtin University	59 (89%)	59 (94%)
University of Western Australia	7 (11%)	4 (6%)
Ethnicity, *n* (%)		
Asian	22 (33%)	18 (29%)
Caucasian	40 (61%)	37 (58%)
Middle Eastern	2 (3%)	3 (5%)
Other	2 (3%)	5 (7%)
Highest level of education completed, *n* (%)		
Master’s degree	2 (3%)	
Graduate Diploma	6 (9%)	2 (3%)
Bachelor’s degree	12 (18%)	15 (23%)
Vocational qualification	9 (14%)	5 (8%)
Year 12 or equivalent	35 (53%)	38 (60%)
Other	2 (3%)	3 (5%)
Course, *n* (%)		
Dentistry	4 (6%)	2 (3%)
Education	12 (18%)	19 (30%)
Medicine	2 (3%)	2 (3%)
Nursing	7 (11%)	3 (5%)
Occupational therapy	17 (26%)	17 (27%)
Pharmacy	5 (8%)	3 (5%)
Psychology	14 (21%)	9 (14%)
Social works	3 (5%)	2 (3%)
Speech pathology	1 (1%)	3 (5%)
Other	1 (1%)	3 (5%)
Study mode, *n* (%)		
Full time	61 (92%)	56 (89%)
Part-time	5 (8%)	7 (11%)
The rank of socioeconomic disadvantage ^a^, *n* (%)		
1–3	3 (5%)	6 (10%)
4–6	26 (39%)	16 (25%)
7–10	35 (56%)	41 (65%)
Previous exposure to a mental health training experience	20 (30%)	16 (25%)
Previous exposure to mental health work experience	6 (9%)	9 (14%)
Living arrangements		
At home with my parents	18 (27%)	25 (39%)
By myself	3 (5%)	3 (5%)
Student dormitory	1 (2%)	3 (5%)
With my partner	11 (17%)	9 (14%)
Other	6 (9%)	11 (17%)

*ATSPPH* Attitudes towards seeking professional psychological help scale, *BRS* Brief resilience scale, *DSG* Delayed start group, *ESG* Early start group, *GSE* General self-efficacy scale, *OSVE* Objective structured video examinations, *PAS* Perception of academic stress scale, *SIDAS* Suicidal ideation attributes scale, *SIRI*-*2* Suicide intervention response inventory 2nd edition

^a^Categorised based on the Australian Bureau of Statistics, with higher scores indicating less disadvantage (www.abs.gov.au)

^b^As measured at baseline (T0)

^c^As measured at 10 weeks (T1)

**Table 2:** Means and standard deviations for primary and secondary outcome measures at each assessment time point

Outcome Measure	Assessment time	ESG (*n* = 66)		DSG (*n* = 64)	
		*M*	SD	*M*	SD
Primary Outcome					
SIRI^a^	T0	70.62	18.42	69.54	8.81
	T1	65.48	16.38	66.14	15.77
	T2	64.86	15.89	64.39	15.71
Secondary Outcomes					
OSVE^b^	T0	15.00	2.96	14.36	2.49
	T1	15.68	3.25	14.56	2.38
	T2	15.75	3.03	15.33	2.85
GSE^b^	T0	30.09	4.12	29.16	4.43
	T1	30.17	30.17	28.50	4.51
	T2	29.65	29.65	28.83	3.86
ATSPPH^b^	T0	13.95	13.95	14.28	2.52
	T1	13.70	13.70	14.25	3.01
	T2	13.88	13.88	13.98	2.57
PAS^b^	T0	54.06	54.06	53.67	7.65
	T1	54.38	54.38	55.02	7.81
	T2	54.48	54.48	55.64	8.08
BRS^b^	T0	18.80	18.80	19.20	4.22
	T1	19.70	19.70	19.61	4.70
	T2	19.48	19.48	19.22	5.36

*ATSPPH* Attitudes towards seeking professional psychological help scale, *BRS* Brief resilience scale, *DSG* Delayed start group, *ESG* Early start group, *GSE* General self-efficacy scale, *OSVE* Objective structured video examinations, *PAS* Perception of academic stress scale, *SIDAS* Suicidal ideation attributes scale, *SIRI-2* Suicide intervention response inventory 2nd edition, *T0* week 0, *T1* week 10, *T2* week 24

^a^Lower score better outcome

^b^Lower score better outcome

^c^Cohen’s d (effect size): 0.2 (small), 0.5 (medium), 0.8 (large)

^***^p <  0.001; *p < 0 .05

### Primary and secondary outcomes

Findings did not demonstrate any significant difference in SIRI-2 scores between ESG and DSG before crossover was applied (*t*(128) = 0.3; *p* = 0.55; < 0.001, Effect size [ES] = 0.05). Although both ESG and DSG had a decrease in SIRI-2 scores, after completing the MOOC, there was no difference between MOOC and treatment as usual after the crossover with the whole data (*F*(2, 254) = 0.39 ; *p* = .68; ES = 0.11).

**Fig. 2:**
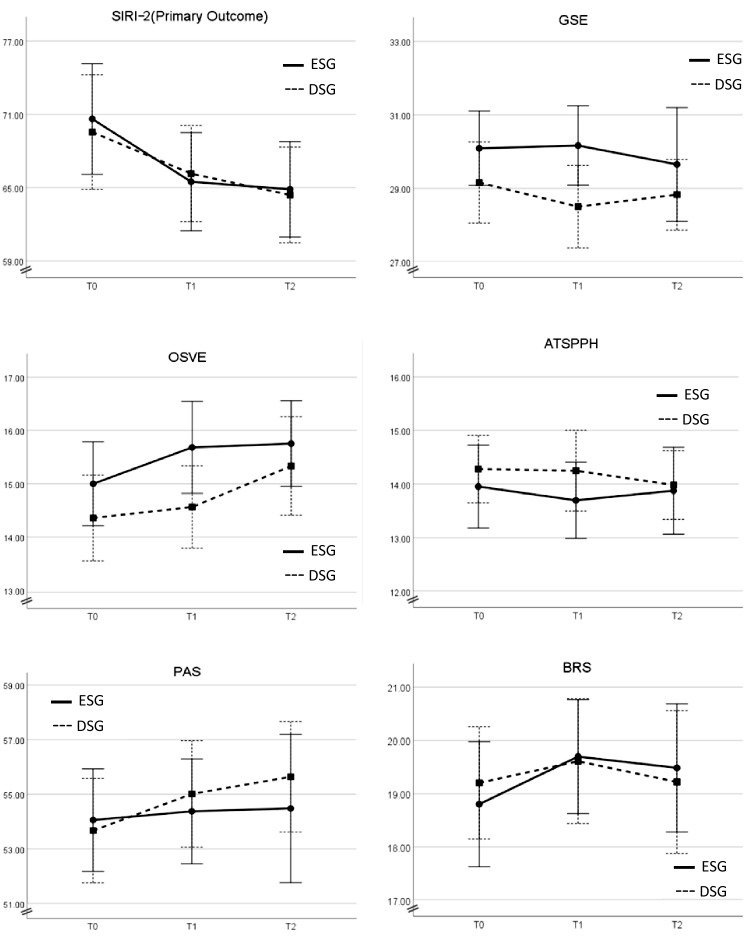
The effects of time by group (ESG/DSG) on the study’s outcome measures. The graphs display the time (T0/T1/T2) by group (ESG/DSG) interaction on the study’s outcome measures, using the random-effects regression model. Errors bars are reported at a 95% confidence interval. *ATSPPH* Attitudes towards seeking professional psychological help scale, *BRS* Brief resilience scale, *DSG* delayed start group, *ESG* early start group, *GSE* General self-efficacy scale, *OSVE* Objective structured video examinations, *PAS* Perception of academic stress scale, *SIRI-2* Suicide intervention response inventory 2nd edition, *T0* week 0, *T1* week 10, *T2* week 24

Similar to SIRI, no significant difference was observed in any of the secondary outcomes between ESG and DSG before or after the crossover of *p* > 0.05, showing small effect sizes (<0.2). The participants’ characteristics and previous experiences showed no moderation effect on these findings (Fig. 2).

Despite the medium and large attrition rates at the end of periods 1 (28%) and 2 (40%), there was no difference between the effect sizes resulting from the ITT approach on any of the outcome measures (primary or secondary) compared to those of the mixed model analysis without any ad-hoc imputation (per-protocol analysis).

### Acceptability of “Talk-to-Me” MOOC

Overall, 74 participants from both the ESG and DSG provided insight into how they perceived the program. Findings suggested a high satisfaction with the content and delivery of the “Talk to Me” MOOC [Median = 43.5, *M *= 41.5 out of 52, SD = 6.2, range = (24, 52)]. Students reported that, on average, they spent 1.44 hours (*SD*=2.27) per module of the “Talk to Me” MOOC. In total, 86% found the content easy to learn, with 73% reporting the program to be very engaging. When asked to nominate the topic that participants found most interesting, 41% chose the topic of mental fitness, followed by strategies to increase mental fitness (39%), self-injury (12%), suicidal behaviour in young adults (5%), and interventions for suicidal behaviour (3%). The results, however, were different when enquiring about the usefulness of the modules, with gatekeeper interventions (24%) and strategies to increase mental fitness (23%) being perceived as more useful, followed by self-injury (20%) and interventions for suicidal behaviour (18%). Mental fitness (4%) and suicidal behaviour in young adults (8%) were considered the least useful. Almost all participants (99%) believed the program had improved their mental health. More than half responded that they could implement the skills and knowledge they learned during the program to improve their own or others’ mental health post completing the program.

## Discussion

This pragmatic crossover RCT evaluated the efficacy of an online psychoeducational Suicide prevention program, “Talk to Me”, firstly in improving university students’ ability to identify and respond to suicidal statements; and, secondly, in improving participants’ knowledge of the program’s content, their ability to recover from stress and adverse events, academic stress, global distress, generalised self-efficacy, and attitudes towards help-seeking behaviour when experiencing mental health issues. Despite the lack of significance for the “Talk to Me” MOOC compared to treatment as usual, in improving the primary and secondary outcomes of this study, students expressed high satisfaction with the program. Although the reason for these contradictory findings is unknown, it highlights the benefits of capturing the student’s views about the program along with the outcome measures [45]. Further, the findings of this study contradict previous research indicating the superiority of a formalised lecture compared to audio-recorded lecture sessions on suicide [46]. As such, demonstrating that utilising extra resources, such as case studies, videos, and quizzes, alone does not support students to apply mental-health-promoting behaviours in their everyday life, contributing to the larger body of knowledge.

Notably, the feasibility and robustness of the outcome framework utilised to measure the efficacy of the MOOC were not assessed prior to the RCT [47]. As such it is unclear whether they were sensitive to capture the true effects of the program. Also, being a self-paced online program, benefitting from the “Talk-to-Me” MOOC mainly relies on the students ‘intrinsic motivation [48]. It is possible that the absence of feedback and structured guidance to students had contributed to the lack of significant finding in this study. Additionally, the commencement of the RCT coincided with the onset of the COVID-19 pandemic, which had an unprecedented impact on the university campus and student life both within Australia and internationally (e.g., widespread campus closures, transitions to online learning) [49]. Notably, during the pandemic, there were other efforts to support the mental well-being of students (e.g., Mental Health First Aid training, online counselling). In addition, in Western Australia, lockdown restrictions were eased and lifted during Semester 2, allowing students to engage in routine daily activities. As such, it is plausible that the observed changes were related to the support students had received during this time.

As discussed in the protocol of the present study [22], based on previous studies employing SIRI-2 as a primary outcome measure [20] and allowing for large attrition of over 100%, researchers aimed to recruit at least 170 participants. It is postulated that the onset of the COVID-19 pandemic negatively impacted study recruitment. A significant number of students were also lost to attrition, and while this reflects the reality of participation in online interventions, it likely had the effect of reducing the power of the study. Notably, the power calculation of the current study was underpinned by a sample simulation. Given the large attrition rate and uncertainty about the student’s level of engagement, the current sample may have been insufficient in capturing the effects of the “Talk-to-Me” MOOC.

Accurately measuring university students’ situational responses to suicidal crises is challenging [17]. A recent scoping review of Suicide prevention programs targeting universities highlighted that outcome measurement frameworks largely rely on assessing changes in students’ help-seeking attitudes and gatekeeper-related outcomes [17]. The impact of these programs on participants’ ability to respond to real-life scenarios remains largely unknown. The present study has been the first to employ an OSVE in assessing the impact of a Suicide prevention program on participants’ behaviour and skills [50]. While the OSVE designed for the present study did not reveal any between-group differences, future research should investigate its utility in measuring student learning outcomes following mental health programs.

Overall, all of the participants who participated in the post-program survey (60%) reported high levels of satisfaction with the “Talk to Me” MOOC content, generally finding the content easy to learn and engaging. It is important that online programs targeting university students are contemporary and enable an emotional connection with participants. A recent systematic review identified intervention-specific and person-specific factors that influence the usage of digital mental health interventions that should be considered. These factors include the medium of delivery mode, language used and helpfulness of the overall program [51]. The “Talk to Me” program was co-produced with individuals with lived experience of mental illness, consulting on areas such as appropriate use of language and usefulness of program content [52]. It is now recognised that the key to the success of any mental health program is developing them in collaboration with the target population [53]. The “Talk to Me” MOOC delivered comprehensive mental health education content, supported in its delivery by two case scenarios of university students experiencing mental health crises. The MOOC was developed in close collaboration with university students, with considerable attention given to ensuring that the program's content was engaging and aligned with the learning preferences of the target group.

Students identified strongly with content relating to mental fitness. Mental fitness is a state of psychological well-being derived from one’s thoughts and emotions and is based on the need for relatedness, competency and autonomy support [54]. Interactions with others tend to act as an enabler or barrier to fulfilling these core psychological needs. When these needs are met within individuals, people experience greater motivation and self-determination in pursuing positive change [54] that could result in higher psychological resilience [55].

Finally, the online delivery of the program provided students with the opportunity to access the material in their own time [56]. The commencement of this study coincided with the onset of the COVID-19 global pandemic and national lockdowns in Australia. It is widely recognised that the onset of the pandemic negatively impacted tertiary students’ mental health [57]. The pandemic raised a critical need for preventative measures capable of reaching students in their home environments [57]. While the findings of the present study are preliminary, they suggest that programs such as the “Talk to Me” MOOC can support tertiary students in these unprecedented times. Future research may benefit from further exploring the efficacy and sustainability of these programs in the long term.

### Limitations and areas for further research

The findings of the current study should be viewed as preliminary in the context of its limitations. This study employed a rigorous crossover design approach to explore the superiority of “Talk to Me” to treatment as usual. However, data collected at the end of Period 1 was used to assess change over the second period, with the carry effects from the first period remaining unknown. Also, as the current study did not collect information about other support or medications the students may have received during their enrollment or employ a methodology enabling daily sampling of students’ mental health during the study period, the effect of other factors influencing the study outcomes remains unknown.

Given the nature and sensitivity of the topic covered in the “Talk-to-Me” MOOC, and according to the ethics recommendations, the researchers were allowed to recruit participants only within their schools, restricting findings to two faculties from two universities situated in Western Australia. Although this limitation provided an opportunity to pilot the program with students whose interests and professional goals aligned with the MOOC, future research may benefit from further exploration of how all university students across Australia may benefit from the “Talk-to-Me” MOOC.

Further, between screening and commencement of the MOOC, participants were required to create a user profile on the edX platform, which may account for the number of dropouts between screening and intervention commencement [58]. Due to data security, this research had access to the edX User statistics only, indicating the number of times a participant watched the videos or the number of times they accessed the modules. This information, however, does not inform the level of engagement of individual participants with the “Talk to Me” content (such as time spent on the platform and engagement with learning activities). As such, it was impossible to determine the true dosage of the intervention. Future studies may consider using research methodologies such as the experiencing sampling method [59] as a way of engaging participants “in situ” to better understand participation and levels of engagement. Although the “Talk-to-Me” MOOC provided real-life scenarios, it could have benefitted from a peer mentor approach. Existing literature indicates that this approach not only supports students through the learning process but also encourages students to translate the newly learned knowledge to their personal lives and promote their connection to campus life [60]. Future studies may investigate how taking this approach would improve outcomes for tertiary students. Finally, working within the university context during the pandemic, where accessing the students in the long term is not possible, this study only captured the maintenance effects 14 weeks from baseline (less than 6 months), with the longer time effects of the program which may take some time to emerge, remaining unknown.

## Conclusions

This study indicated that receiving the online “Talk to Me” MOOC it is not enough to enable students to develop skills to respond to others in distress. Future suicide prevention interventions among tertiary students may consider using online peer mentoring programs to create user groups where participants can practice their skills face-to-face. Further research with a large national sample of university students is also warranted to determine the robustness of the current findings.

### Supplementary Information

Below is the link to the electronic supplementary material.

Supplementary file1 (DOCX 16 KB)

Supplementary file2 (DOCX 18 KB)

Supplementary file3 (DOCX 21 KB)

Supplementary file4 (DOCX 20 KB)

Supplementary file5 (DOCX 27 KB)

## Data Availability

There is no public access to the datasets generated and/or analysed during the current study, and they are only available from the corresponding author upon reasonable request.
