# Supplementary material for: The efficacy of the “Talk-to-Me” suicide prevention and mental health education program for tertiary students: a crossover randomised control trial
Source: Eur Child Adolesc Psychiatry. 2022 Oct 4;32(12):2477–89. doi: 10.1007/s00787-022-02094-4 (PMC9531217; doi:10.1007/s00787-022-02094-4)
Supplement: Supplementary file 1 — Supplementary file1 (DOCX 16 KB) [file 787_2022_2094_MOESM1_ESM.docx]

# Online Resource 1 - Overview of the Talk-to-Me” MOOC

# The “Talk-to-Me” MOOC intervention for suicide prevention and mental health education among tertiary students: A multi-site crossover randomised control trial

# *European Child and Adolescent Psychiatry*

Dr Bahareh Afsharnejad; Dr Ben Milbourn ^a^; Ms Maya Hayden-Evans; Ms Ellie Baker-Young; Dr Melissa H Black; Dr Craig Thompson; Dr Sarah McGarry; Dr Melissa Grobler; Prof. Rhonda Clifford; Mr Frank Zimmermann; Dr Viktor Kacic; Assoc. Prof. Penelope Hasking; Prof. Sven Bölte; Prof. Marcel Romanos; Assis. Prof. Tawanda Machingura; Prof. Sonya Girdler..

^a^ Corresponding author: School of Allied Health, Curtin University, Perth, Western Australia; Curtin Autism Research Group (CARG), Curtin University, Perth, Western Australia; enAble Institute, Curtin University, Perth, Western Australia; Ben.milbourn@curtin.edu.au

**Table A1.** Overview of the “Talk-to-Me” MOOC content

| **Module** | **Topic** | **Module Content** |
| --- | --- | --- |
| **1** | Mental fitness | Defining mental health, its theories and statistics; increasing positive mental health through Act-Belong-Commit activities; utilising the interactive visual “traffic light system” to inform the level of mental wellbeing; providing case study video scenarios of a regional student’s experience at university; teaching stress management strategies; delivering interactive “Talk-to-Me” questionnaire; and, testing participants understanding of the content. |
| **2** | Strategies to increase mental fitness | Utilising metacognitive strategies and video visual metaphors for stress management; teaching the ABC model; completing interactive worksheets about negative thought patterns; providing case study video scenarios, showing how one can respond to poor mental health suicidal ideation; testing participants understanding of the content. |
| **3** | Self-injury | Defining deliberate self-harm and non-suicidal self-Injury through text and video and how it might be used as a coping strategy; learning how to respond to self-harm concerns with examples and support someone in crisis; teaching emotional regulation strategies, safety planning, with examples; testing participants understanding of the content. |
| **4** | Suicidal behaviour in young adults | Explaining risk factors associated with suicidal behaviour in young adults; case study video scenarios modelling appropriate responses to suicidal ideation; enquiring about risk factors via an interactive questionnaire; testing participants understanding of the content. |
| **5** | Interventions for suicidal behaviour | Providing a suicide prevention plan; case study video scenarios; describing decision tree for handling suicidality; practising mindfulness exercises; describing general methods of suicide prevention; explaining how applying the ‘Talk-to-Me’ program principles can be used in an emergency; testing participants understanding of the content. |
| **6** | Gatekeeper interventions | Explaining mental illness and suicide facts and myths; case study video scenarios; managing emergencies and responding to a mental health crisis; describing self-care plan with examples; facilitating appropriate collaborative care; practising grounding exercises; testing participants understanding of the content. |
