# Supplementary material for: The efficacy of the “Talk-to-Me” suicide prevention and mental health education program for tertiary students: a crossover randomised control trial
Source: Eur Child Adolesc Psychiatry. 2022 Oct 4;32(12):2477–89. doi: 10.1007/s00787-022-02094-4 (PMC9531217; doi:10.1007/s00787-022-02094-4)
Supplement: Supplementary file 2 — Supplementary file2 (DOCX 18 KB) [file 787_2022_2094_MOESM2_ESM.docx]

# Online Resource 2 - Overview of the OSVE Measure

# The “Talk-to-Me” MOOC intervention for suicide prevention and mental health education among tertiary students: A multi-site crossover randomised control trial

# *European Child and Adolescent Psychiatry*

Dr Bahareh Afsharnejad; Dr Ben Milbourn ^a^, Ms Maya Hayden-Evans; Ms Ellie Baker-Young; Dr Melissa H Black, Dr Craig Thompson; Dr Sarah McGarry; Dr Melissa Grobler; Prof. Rhonda Clifford; Mr Frank Zimmermann; Dr Viktor Kacic; Assoc. Prof. Penelope Hasking; Prof. Sven Bölte; Prof. Marcel Romanos; Assis. Prof. Tawanda Machingura; Prof. Sonya Girdler^a^ Corresponding author: School of Allied Health, Curtin University, Perth, Western Australia; Curtin Autism Research Group (CARG), Curtin University, Perth, Western Australia; enAble Institute, Curtin University, Perth, Western Australia; [Ben.milbourn@curtin.edu.au](mailto:Ben.milbourn@curtin.edu.au)

**Table A2.** The Overview of the OSVE Measure in Relation to the “Talk-to-Me” Modules

| Items | Category | MOOC module |
| --- | --- | --- |
| Video 1 | | |
| 1. Which of the following are ALL suicide risk factors for Mike? | Identifying risk factors and warning signs | 1,4,5 |
| 2. Which of the following are ALL suicide warning signs for Mike? | Identifying risk factors and warning signs | 1,4,5 |
| 3. Which of the following statements demonstrate 'irrational' unhelpful thinking by Mike? | identifying unhelpful thoughts | 2 |
| 4. What could Corey say to Mike that would appropriately challenge the unhelpful thinking? | Challenging unhelpful thoughts | 2 |
| 5. "I'll probably never play hockey again." is an example of which irrational thinking error? | Identifying unhelpful thoughts | 2 |
| Video 2 | | |
| 1. Why did Mike punch the wall? | Types of coping strategies for handling stress | 1 |
| 2. Corey's response "You don't need to do that! You're mental." is an example of: | how to respond to non-suicidal self-harm concerns | 3 |
| 3. What would be an appropriate response from Corey? | how to respond to non-suicidal self-harm concerns | 3 |
| 4. Corey's offer to hook Mike up with someone else is an example of what? | Alternative response to non-suicidal self-harm | 3 |
| 5. What are some examples of healthy coping strategies that Corey could suggest to Mike? | Types of coping strategies for handling stress | 1,3 |
| Video 3 | | |
| 1. Which statements by Mike most strongly indicate he could be feeling suicidal? | Identifying risk factors or triggers for suicidal ideation | 4 |
| 2. What could Corey ask next to appropriately gauge Mike's level of suicide risk? | Conducting a suicide risk assessment | 4,5 |
| 3. Mike's uncle's death by suicide is an example of: | Identifying risk factors or triggers for suicidal ideation | 4 |
| 4. Which of the following stressors is contributing to Mike's suicidal feelings? | Identifying risk factors or triggers for suicidal ideation | 4 |
| 5. What could Corey ask to appropriately assess the presence of protective factors for Mike? | Conducting a suicide risk assessment | 4,5 |
| Video 4 | | |
| 1. In this situation, Josh's relationship to Mike is that of a: | Handling an emergency | 5,6 |
| 2. What tone of voice and pace of speaking should Josh use during this conversation? | Alternative response to suicidal ideation | 3,6 |
| 3. What coping strategy was Josh suggesting to Mike to study? | Alternative response to suicidal ideation | 3,6 |
| 4. Josh’s "trick" for improved concentration for study is an example of: | Mindfulness activities | 2,3,6 |
| 5. What statements should Josh avoid in this conversation? | How to talk to someone with Suicidal ideation | 3,6 |
| video 5 | | |
| 1. Josh is demonstrating which of the following non-verbal elements of active listening: | Active listening skills | 2,3,4,5,6 |
| 2. Josh asking, "have you thought about how you'd do it?" and "do you have a timeline?" are examples of: | Conducting a suicide risk assessment | 4,5 |
| 3. Which factors are signs of imminent risk of suicide for Mike? | Conducting a suicide risk assessment | 4,6 |
| 4. What are the 3 key components Josh needs to create a safety plan for Mike? | Safety Planning | 3 |
| 5. How should Josh conclude this conversation? | Handling an emergency | 5,6 |
