# Supplementary material for: The efficacy of the “Talk-to-Me” suicide prevention and mental health education program for tertiary students: a crossover randomised control trial
Source: Eur Child Adolesc Psychiatry. 2022 Oct 4;32(12):2477–89. doi: 10.1007/s00787-022-02094-4 (PMC9531217; doi:10.1007/s00787-022-02094-4)
Supplement: Supplementary file 3 — Supplementary file3 (DOCX 21 KB) [file 787_2022_2094_MOESM3_ESM.docx]

# Online Resource 3 - "Talk to Me" MOOC Evaluation Questionnaire

# The “Talk-to-Me” MOOC intervention for suicide prevention and mental health education among tertiary students: A multi-site crossover randomised control trial

# *European Child and Adolescent Psychiatry*

Dr Bahareh Afsharnejad; Dr Ben Milbourn ^a^; Ms Maya Hayden-Evans; Ms Ellie Baker-Young; Dr Melissa H Black; Dr Craig Thompson; Dr Sarah McGarry; Dr Melissa Grobler; Prof. Rhonda Clifford; Mr Frank Zimmermann; Dr Viktor Kacic; Assoc. Prof. Penelope Hasking; Prof. Sven Bölte; Prof. Marcel Romanos; Assis. Prof. Tawanda Machingura; Prof. Sonya Girdler.

^a^ Corresponding author: School of Allied Health, Curtin University, Perth, Western Australia; Curtin Autism Research Group (CARG), Curtin University, Perth, Western Australia; enAble Institute, Curtin University, Perth, Western Australia; Ben.milbourn@curtin.edu.au

These questions are about your use of the "Talk to Me" MOOC (Mass Open Online Course). Please read the items and tell us how you felt about the MOOC. We encourage students be completely honest with their response - we would like to know what you really think to help us improve the MOOC for future students.  If the item does not apply, please choose “NA”.

| **SECTION 1 - OPEN-ENDED QUESTIONS** |
| --- |

| 1. Approximately how long did each module take to complete? |
| --- |
| 2. How easy was it to complete the amount of content in the MOOC within the specified time-frame? (1- 2hrs per module). |
| 3. Which modules did you find interesting? Tick all that apply.  ⃝ 1 ⃝ 2 ⃝ 3 ⃝ 4 ⃝ 5 ⃝ 6 |
| 4. What was the most useful topic of the MOOC and why? |
| 5. Did you feel your mental health skills/knowledge improved as a result of the MOOC?  ⃝ Yes  ⃝ No (optional - You may specify below) |
| 6. Have you implemented any of the skills/knowledge learned to help improve your own or others' mental health since completing the MOOC?  ⃝ No  ⃝ Yes (optional - You may include an example below) |
| What improvements would you recommend for the MOOC? |
| Do you have any additional comments you would like to provide about your experience with the MOOC?  ⃝ Yes ⃝ No |

| **SECTION 2 - SATISFACTION** |
| --- |

We included a range of different learning resources within each module and want to find out which resources assisted you to engage with the course and understand key concepts. Please rate the helpfulness of different types of resources using the scale below.

|  | Not helpful at all | A little helpful | Very helpful | Extremely helpful |
| --- | --- | --- | --- | --- |
| Intro videos to modules | ⃝ | ⃝ | ⃝ | ⃝ |
| Case study roleplay videos (e.g., Ken and Charlotte) | ⃝ | ⃝ | ⃝ | ⃝ |
| YouTube Videos (external) | ⃝ | ⃝ | ⃝ | ⃝ |
| Questionnaires (e.g., 'Talk to Me' questionnaire, Risk assessment) | ⃝ | ⃝ | ⃝ | ⃝ |
| Links to further readings or references (websites, articles) | ⃝ | ⃝ | ⃝ | ⃝ |
| Mindfulness exercises | ⃝ | ⃝ | ⃝ | ⃝ |

How engaging did you find the MOOC?

⃝ Extremely engaging

⃝ Very engaging

⃝ Engaging

⃝ Not engaging

The MOOC covered a range of topics designed to help improve mental health knowledge and skills to respond to mental health crises, as well as support your own or others' mental health. Please rate the helpfulness of the topics covered in the course. You may also rate how well you thought each topic was covered in the course. For example, if you felt a more information was required for a topic- select "Needed elaboration".

|  | Not helpful at all | A little helpful | Very helpful | Extremely helpful | Needed Elaboration | Sufficiently covered | Needed less elaboration |
| --- | --- | --- | --- | --- | --- | --- | --- |
| Mental health education (e.g., statistics, definitions of key concepts) | ⃝ | ⃝ | ⃝ | ⃝ | ⃝ | ⃝ | ⃝ |
| Crisis communication skills - responding to suicide | ⃝ | ⃝ | ⃝ | ⃝ | ⃝ | ⃝ | ⃝ |
| Crisis communication skills - responding to suicide | ⃝ | ⃝ | ⃝ | ⃝ | ⃝ | ⃝ | ⃝ |
| Crisis communication skills - responding to self-harm | ⃝ | ⃝ | ⃝ | ⃝ | ⃝ | ⃝ | ⃝ |
| Coping strategies for suicidal ideation | ⃝ | ⃝ | ⃝ | ⃝ | ⃝ | ⃝ | ⃝ |
| Coping strategies for self-harm | ⃝ | ⃝ | ⃝ | ⃝ | ⃝ | ⃝ | ⃝ |
| Strategies to improve mental fitness (e.g., cognitive restructuring, mindfulness) | ⃝ | ⃝ | ⃝ | ⃝ | ⃝ | ⃝ | ⃝ |
| Safety Planning | ⃝ | ⃝ | ⃝ | ⃝ | ⃝ | ⃝ | ⃝ |
| Managing mental health emergencies | ⃝ | ⃝ | ⃝ | ⃝ | ⃝ | ⃝ | ⃝ |
