# Supplementary material for: The efficacy of the “Talk-to-Me” suicide prevention and mental health education program for tertiary students: a crossover randomised control trial
Source: Eur Child Adolesc Psychiatry. 2022 Oct 4;32(12):2477–89. doi: 10.1007/s00787-022-02094-4 (PMC9531217; doi:10.1007/s00787-022-02094-4)
Supplement: Supplementary file 4 — Supplementary file4 (DOCX 20 KB) [file 787_2022_2094_MOESM4_ESM.docx]

# Online Resource 4 - COVID-19 Questionnaire

# The “Talk-to-Me” MOOC intervention for suicide prevention and mental health education among tertiary students: A multi-site crossover randomised control trial

# *European Child and Adolescent Psychiatry*

Dr Bahareh Afsharnejad; Dr Ben Milbourn ^a^; Ms Maya Hayden-Evans; Ms Ellie Baker-Young; Dr Melissa H Black; Dr Craig Thompson; Dr Sarah McGarry; Dr Melissa Grobler; Prof. Rhonda Clifford; Mr Frank Zimmermann; Dr Viktor Kacic; Assoc. Prof. Penelope Hasking; Prof. Sven Bölte; Prof. Marcel Romanos; Assis. Prof. Tawanda Machingura; Prof. Sonya Girdler.

^a^ Corresponding author: School of Allied Health, Curtin University, Perth, Western Australia; Curtin Autism Research Group (CARG), Curtin University, Perth, Western Australia; enAble Institute, Curtin University, Perth, Western Australia; Ben.milbourn@curtin.edu.au

Please read the questions and tell us how you have felt since the COVID-19 crisis. We encourage students to be completely honest with their responses - we would like to know what you really think. Your feedback will help us to understand the impact of COVID-19 and the need for the MOOC for future students.

| I was diagnosed with a mental health diagnosis during the COVID 19 crisis | ⃝ Yes | ⃝ No |
| --- | --- | --- |
| I received treatment for my mental health during the COVID 19 crisis | ⃝ Yes | ⃝ No |
| A friend/partner/family member has been infected with COVID 19 | ⃝ Yes | ⃝ No |

| **#** | **Since COVID 19...** | Never | Sometimes | Often | Always |
| --- | --- | --- | --- | --- | --- |
| 1 | ... I generally feel more burdened | ⃝ | ⃝ | ⃝ | ⃝ |
| 2 | ...I have become annoyed even over little things | ⃝ | ⃝ | ⃝ | ⃝ |
| 3 | ... I have more fears than usual | ⃝ | ⃝ | ⃝ | ⃝ |
| 4 | ... I look up more topics of self-harm on the internet | ⃝ | ⃝ | ⃝ | ⃝ |
| 5 | ... I stick to hygiene and distance rules | ⃝ | ⃝ | ⃝ | ⃝ |
| 6 | ... I think that the health of other people is more important than my health problems | ⃝ | ⃝ | ⃝ | ⃝ |
| 7 | ... I am often bored | ⃝ | ⃝ | ⃝ | ⃝ |
| 8 | ... I feel helpless | ⃝ | ⃝ | ⃝ | ⃝ |
| 9 | ...I feel as relaxed as usual | ⃝ | ⃝ | ⃝ | ⃝ |
| 10 | ... I am careful not to get infected with COVID 19 | ⃝ | ⃝ | ⃝ | ⃝ |
| 11 | ... I have an organised daily structure | ⃝ | ⃝ | ⃝ | ⃝ |
| 12 | ... I realise the longer the crisis is lasting the more difficult it will be to cope with everyday life | ⃝ | ⃝ | ⃝ | ⃝ |
| 13 | ... I am no longer interested in activities that I previously enjoyed | ⃝ | ⃝ | ⃝ | ⃝ |
| 14 | ... I am worried about the future | ⃝ | ⃝ | ⃝ | ⃝ |
| 15 | ... I put myself in danger because of my risky behaviour | ⃝ | ⃝ | ⃝ | ⃝ |
| 16 | ... I do not have a close person to talk to | ⃝ | ⃝ | ⃝ | ⃝ |
| 17 | ... There is help available if I need it | ⃝ | ⃝ | ⃝ | ⃝ |
| 18 | ... I can cope with the COVID 19 restrictions | ⃝ | ⃝ | ⃝ | ⃝ |
| 19 | ... I find it difficult to study | ⃝ | ⃝ | ⃝ | ⃝ |
| 20 | ...I think my university education will be successful | ⃝ | ⃝ | ⃝ | ⃝ |
| 21 | ... I drink more alcohol/ use more drugs | ⃝ | ⃝ | ⃝ | ⃝ |
| 22 | ... I am overwhelmed with the news about COVID 19 | ⃝ | ⃝ | ⃝ | ⃝ |
| 23 | ... I think that I have to solve all my problems by myself | ⃝ | ⃝ | ⃝ | ⃝ |
| 24 | ... I continue to maintain my social contacts | ⃝ | ⃝ | ⃝ | ⃝ |
| 25 | ... I am continuing to sleep well | ⃝ | ⃝ | ⃝ | ⃝ |
| 26 | ... I avoid leaving the house if possible | ⃝ | ⃝ | ⃝ | ⃝ |
| 27 | ... I am creative in my leisure activities | ⃝ | ⃝ | ⃝ | ⃝ |
| 28 | ... I know who to talk to if I do not feel well | ⃝ | ⃝ | ⃝ | ⃝ |

Scoring:

Score Items 5, 9, 10, 11, 17, 18, 20, 24, 25, 27, 28 as follows: Never = 0; Sometimes = 1; Often = 2; Always = 3

Score Items 5, 9, 10, 11, 17, 18, 20, 24, 25, 27, 28 as follows: Never = 3; Sometimes = 2; Often = 1; Always = 0
