# Supplementary material for: The efficacy of the “Talk-to-Me” suicide prevention and mental health education program for tertiary students: a crossover randomised control trial
Source: Eur Child Adolesc Psychiatry. 2022 Oct 4;32(12):2477–89. doi: 10.1007/s00787-022-02094-4 (PMC9531217; doi:10.1007/s00787-022-02094-4)
Supplement: Supplementary file 5 — Supplementary file5 (DOCX 27 KB) [file 787_2022_2094_MOESM5_ESM.docx]

# Online Resource 5 - Detailed Results

# The “Talk-to-Me” MOOC intervention for suicide prevention and mental health education among tertiary students: A multi-site crossover randomised control trial

# *European Child and Adolescent Psychiatry*

Dr Bahareh Afsharnejad; Dr Ben Milbourn ^a^, Ms Maya Hayden-Evans; Ms Ellie Baker-Young; Dr Melissa H Black, Dr Craig Thompson; Dr Sarah McGarry; Dr Melissa Grobler; Prof. Rhonda Clifford; Mr Frank Zimmermann; Dr Viktor Kacic; Assoc. Prof. Penelope Hasking; Prof. Sven Bölte; Prof. Marcel Romanos; Assis. Prof. Tawanda Machingura; Prof. Sonya Girdler.

^a^ Corresponding author: School of Allied Health, Curtin University, Perth, Western Australia; Curtin Autism Research Group (CARG), Curtin University, Perth, Western Australia; enAble Institute, Curtin University, Perth, Western Australia; [Ben.milbourn@curtin.edu.au](mailto:Ben.milbourn@curtin.edu.au)

**Table A3** Outcomes measures at Times 0, 1, and 2: Means, standard deviations (SD), between/within group differences (including comparisons, mean difference, probability values (p values) for comparisons, effect size and 95% confidence intervals

| Measure | | Mean (SD) | |  | Between groups | | | |  | Within group | | | | | | | |
| --- | --- | --- | --- | --- | --- | --- | --- | --- | --- | --- | --- | --- | --- | --- | --- | --- | --- |
|  |  |  |  |  |  |  |  |  |  | ESG | | | | DSG | | | |
|  |  | ESG | DSG |  | M_Diff | *p* | ES ^c^ | 95% CI | Comparison | M_Diff | *p* | ES ^c^ | 95% CI | M_Diff | *p* | ES ^c^ | 95% CI |
| Primary outcome | | | | | | | | | | | | | | | | | |
| SIRI^a^ | T0 | 70.62 (18.42) | 69.54 (18.81) |  |  |  |  |  | T0 to T1 | 5.14* | <.001 | 0.29 | [1.85, 8.43] | 3.40* | .04 | 0.20 | [0.06, 6.74] |
|  | T1 | 65.48 (16.38) | 66.14 (15.77) |  | -0.67 | 0.82 | 0.04 | [-6.51, 5.18] | T0 to T2 | 5.76* | <.001 | 0.34 | [2.48, 9.05] | 5.15* | <.001 | 0.30 | [1.82, 8.49] |
|  | T2 | 64.86 (15.89) | 64.39 (15.71) |  | 0.47 | 0.88 | 0.03 | [-5.38, 6.31] | T1 to T2 | 0.62 | 1.00 | 0.04 | [-2.67, 3.91] | 1.75 | .62 | 0.11 | [-1.58, 5.09] |
| Secondary Outcomes | | | | | | | | | | | | | | | | | |
| GSE^b^ | T0 | 30.09 (4.12) | 29.16 (4.43) |  |  |  |  |  | T0 to T1 | -0.08 | 1.00 | 0.02 | [-1.22, 1.07] | 0.66 | .52 | 0.15 | [-0.51, 1.82] |
|  | T1 | 30.17 (4.40) | 28.50 (4.51) |  | 1.67* | 0.04 | 0.37 | [0.05, 3.28] | T0 to T2 | 0.44 | 1.00 | 0.08 | [-0.7, 1.58] | 0.33 | 1.00 | 0.08 | [-0.83, 1.49] |
|  | T2 | 29.65 (6.30) | 28.83 (3.86) |  | 0.82 | 0.32 | 0.16 | [-0.79, 2.44] | T1 to T2 | 0.52 | 0.84 | 0.09 | [-0.63, 1.66] | -0.33 | 1.00 | 0.08 | [-1.49, 0.83] |
| OSVE^b^ | T0 | 15.00 (2.96) | 14.36 (2.49) |  |  |  |  |  | T0 to T1 | -0.68* | .04 | 0.22 | [-1.36, -0.01] | -0.21 | 1.00 | 0.08 | [-1.02, 0.61] |
|  | T1 | 15.68 (3.25) | 14.56 (2.38) |  | 1.12 | 0.06 | 0.38 | [-0.07, 2.31] | T0 to T2 | -0.75* | .02 | 0.25 | [-1.43, -0.08] | -0.97* | .01 | 0.36 | [-1.79, -0.16] |
|  | T2 | 15.75 (3.03) | 15.33 (2.85) |  | 0.42 | 0.49 | 0.14 | [-0.77, 1.61] | T1 to T2 | -0.07 | 1.00 | 0.02 | [-0.74, 0.6] | -0.77 | .07 | 0.29 | [-1.58, 0.04] |
| ATSPPH^b^ | T0 | 13.95 (3.14) | 14.28 (2.52) |  |  |  |  |  | T0 to T1 | 0.26 | 1.00 | 0.09 | [-0.56, 1.07] | 0.03 | 1.00 | 0.01 | [-0.8, 0.86] |
|  | T1 | 13.7 (2.89) | 14.25 (3.01) |  | -0.55 | .28 | 0.19 | [-1.56, 0.46] | T0 to T2 | 0.08 | 1.00 | 0.02 | [-0.74, 0.89] | 0.3 | 1.00 | 0.12 | [-0.53, 1.13] |
|  | T2 | 13.88 (3.3) | 13.98 (2.57) |  | -0.11 | .84 | 0.04 | [-1.12, 0.91] | T1 to T2 | -0.18 | 1.00 | 0.06 | [-1.00, 0.63] | 0.27 | 1.00 | 0.1 | [-0.56, 1.09] |
| PAS^b^ | T0 | 54.06 (7.62) | 53.67 (7.65) |  |  |  |  |  | T0 to T1 | -0.32 | 1.00 | 0.04 | [-2.53, 1.89] | -1.34 | .45 | 0.17 | [-3.59, 0.9] |
|  | T1 | 54.38 (7.81) | 55.02 (7.81) |  | -0.64 | .67 | 0.08 | [-3.55, 2.28] | T0 to T2 | -0.42 | 1.00 | 0.04 | [-2.63, 1.78] | -1.97 | .11 | 0.25 | [-4.21, 0.27] |
|  | T2 | 54.48 (11.05) | 55.64 (8.08) |  | -1.16 | .44 | 0.12 | [-4.07, 1.76] | T1 to T2 | -0.11 | 1.00 | 0.01 | [-2.31, 2.1] | -0.63 | 1.00 | 0.08 | [-2.87, 1.62] |
| BRS^b^ | T0 | 18.8 (4.78) | 19.2 (4.22) |  |  |  |  |  | T0 to T1 | -0.89 | .43 | 0.2 | [-2.36, 0.57] | -0.41 | 1.00 | 0.09 | [-1.89, 1.08] |
|  | T1 | 19.7 (4.35) | 19.61 (4.7) |  | 0.09 | .92 | 0.02 | [-1.55, 1.72] | T0 to T2 | -0.68 | .79 | 0.14 | [-2.15, 0.78] | -0.02 | 1.00 | 0 | [-1.5, 1.47] |
|  | T2 | 19.48 (4.9) | 19.22 (5.36) |  | 0.27 | .75 | 0.05 | [-1.37, 1.9] | T1 to T2 | 0.21 | 1.00 | 0.05 | [-1.25, 1.68] | 0.39 | 1.00 | 0.08 | [-1.1, 1.88] |

Note: ATSPPH = Attitudes Towards Seeking Professional Psychological Help Scale; BRS = Brief Resilience Scale; CI = confidence interval; DSG = delayed start group; ESG = early start group; ES = effect size; GSE = General Self-Efficacy scale; M-Diff = mean difference; OSVE = Objective Structured Video Examinations; PAS = Perception of Academic Stress Scale; SIDAS = Suicidal Ideation Attributes Scale; SIRI-2 = Suicide Intervention Response Inventory 2nd edition, T0 = week 0; T1 = week 10; T2 = week 24.

^a^ Lower score = better outcome; ^b^ Lower score = better outcome; ^c^ Cohen’s d (effect size): .2 (small), .5 (medium), .8 (large).

^***^ *p* < .001; ^*^ *p* < .05
